# Biomimetic Electrospun Scaffold-Based In Vitro Model Resembling the Hallmarks of Human Myocardial Fibrotic Tissue

**DOI:** 10.1021/acsbiomaterials.3c00483

**Published:** 2023-06-08

**Authors:** Gerardina Ruocco, Alice Zoso, Leonardo Mortati, Irene Carmagnola, Valeria Chiono

**Affiliations:** † Department of Mechanical and Aerospace Engineering, Politecnico di Torino, 10129 Torino TO, Italy; ‡ POLITO Biomedlab, Politecnico di Torino, 10129 Torino TO, Italy; § Interuniversity Center for the Promotion of the 3Rs Principles in Teaching and Research, 56122 Pisa PI, Italy; ∥ Istituto Nazionale di Ricerca Metrologica (INRIM), 10135 Torino TO, Italy

**Keywords:** electrospun scaffold, surface modification, *in vitro* model, cardiac fibrosis, extracellular matrix proteins

## Abstract

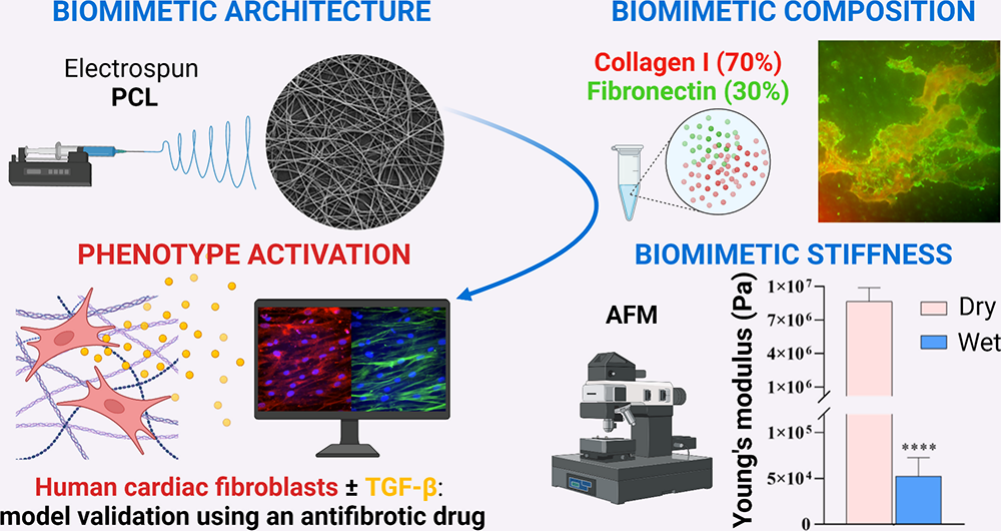

Adverse remodeling post-myocardial infarction is hallmarked by the phenotypic change of cardiac fibroblasts (CFs) into myofibroblasts (MyoFs) and over-deposition of the fibrotic extracellular matrix (ECM) mainly composed by fibronectin and collagens, with the loss of tissue anisotropy and tissue stiffening. Reversing cardiac fibrosis represents a key challenge in cardiac regenerative medicine. Reliable *in vitro* models of human cardiac fibrotic tissue could be useful for preclinical testing of new advanced therapies, addressing the limited predictivity of traditional 2D cell cultures and animal *in vivo* models. In this work, we engineered a biomimetic *in vitro* model, reproducing the morphological, mechanical, and chemical cues of native cardiac fibrotic tissue. Polycaprolactone (PCL)-based scaffolds with randomly oriented fibers were fabricated by solution electrospinning technique, showing homogeneous nanofibers with an average size of 131 ± 39 nm. PCL scaffolds were then surface-functionalized with human type I collagen (C1) and fibronectin (F) by dihydroxyphenylalanine (DOPA)-mediated mussel-inspired approach (PCL/polyDOPA/C1F), in order to mimic fibrotic cardiac tissue-like ECM composition and support human CF culture. BCA assay confirmed the successful deposition of the biomimetic coating and its stability during 5 days of incubation in phosphate-buffered saline. Immunostaining for C1 and F demonstrated their homogeneous distribution in the coating. AFM mechanical characterization showed that PCL/polyDOPA/C1F scaffolds, in wet conditions, resembled fibrotic tissue stiffness with an average Young’s modulus of about 50 kPa. PCL/polyDOPA/C1F membranes supported human CF (HCF) adhesion and proliferation. Immunostaining for α-SMA and quantification of α-SMA-positive cells showed HCF activation into MyoFs in the absence of a transforming growth factor β (TGF-β) profibrotic stimulus, suggesting the intrinsic ability of biomimetic PCL/polyDOPA/C1F scaffolds to sustain the development of cardiac fibrotic tissue. A proof-of-concept study making use of a commercially available antifibrotic drug confirmed the potentialities of the developed *in vitro* model for drug efficacy testing. In conclusion, the proposed model was able to replicate the main hallmarks of early-stage cardiac fibrosis, appearing as a promising tool for future preclinical testing of advanced regenerative therapies.

## 1 Introduction

Heart failure (HF) represents a huge global burden, affecting around 64 million people worldwide.^1^ HF arises as a consequence of cardiac fibrosis, a common pathophysiological mechanism in most cardiac diseases such as myocardial infarction (MI), and leads to impaired cardiac function and arrhythmias.^2,3^ Fibrotic remodeling starts as a reparative response triggered by the loss of cardiomyocytes (CMs) and proceeds through a wound-healing process aimed at preserving the tissue structural integrity.^4^ In the early inflammatory phase of replacement fibrosis, a dynamic extracellular matrix containing fibrin and fibronectin is formed as a provisional scaffold for tissue healing.^5^ In the subsequent proliferative phase, upon mechanical or biochemical stimuli, cardiac fibroblasts (CFs) undergo a phenotypic change into myofibroblasts (MyoFs). MyoFs express high levels of contractile proteins, such as alpha-smooth muscle actin (α-SMA), vimentin, and focal adhesion proteins, allowing the contraction of the surrounding extracellular matrix (ECM) and scar formation.^6−8^ The acquisition of a mature myofibroblast phenotype is mediated by an increased secretion of anti-inflammatory and profibrotic factors, such as angiotensin II (Ang II) and transforming growth factor-β (TGF-β), by both infiltrating immune and myocardial cells.^9,10^ TGF-β inhibits the synthesis of metalloproteinases, suppressing ECM degradation, and triggers the deposition of ECM proteins by MyoFs, namely collagen types I, III, IV, V, and VI (with collagen types I and III as the major components of the scar tissue), fibronectin, laminin α1, and tenascin-X.^11−13^ The increase in ECM protein concentration is accompanied by progressive architecture disruption, which causes CM hypertrophy and anisotropy loss. As a consequence, tissue stiffness increases from 10 kPa for healthy cardiac tissue to 30–100 kPa for pathological cardiac tissue.^14^

Heart transplantation still remains the only clinical option to treat end-stage HF, although limited by the lack of donors and the risks associated with immunosuppression. Hence, effective therapies for cardiac fibrosis regression and heart functional recovery are highly demanded. Advanced approaches for cardiac regeneration are under study and include cell reprogramming strategies^15,16^ aimed at inducing cardiomyocyte proliferation or the trans-differentiation of CFs into cardiomyocytes, and stem cell therapies.^17^

Preclinical validation of new advanced approaches/drugs could benefit from reliable *in vitro* models of human cardiac tissue, in agreement with the 3Rs principle (Reduction, Replacement, and Refinement).^18^ Indeed, *in vitro* human tissue models provide relevant testing platforms, able to overcome the poor tissue specificity of traditional 2D cultures, as well as the limited predictivity and ethical issues of in vivo animal models.

Until now, *in vitro* models of pathological cardiac tissue have been mainly engineered making use of hydrogel substrates with tissue-like stiffness. Zhao et al.^19^ studied an *in vitro* model of cardiac fibrosis, based on polyethylene glycol diacrylate (PEGDA) hydrogel with patterned stiffness, functionalized with collagen and cellularized with adult rat CFs. The PEGDA/collagen hydrogel promoted adult rat CF activation, with CF differentiation into MyoFs on stiff (∼40 kPa) substrates. *In vitro* studies on this model evidenced that increasing amounts of Rho-associated protein kinase (ROCK) inhibitor reduced the number of MyoFs. Deddens et al.^20^ engineered an *in vitro* model of fibrosis by encapsulating rat adult CFs in a collagen-based hydrogel. The study aimed at measuring the effect of a thrombin receptor inhibitor (PAR1) on cardiac fibrosis. Thrombin addition significantly increased tissue stiffness, which was then reduced by the administration of PAR1 inhibitor. This work underlined the role of a pathological *in vitro* model in the identification of drug targets and the pathophysiological processes with a key role on cardiac fibrosis and adverse ventricular remodeling. Sadeghi et al.^21^ developed 3D gelatin–methacryloyl (GelMA) hydrogels with different stiffnesses, co-cultured with neonatal rat CMs and CFs. The expression of α-SMA, collagen type I, fibronectin, and matrix metalloproteinase-2 (MMP-2) confirmed the CF activation into MyoFs upon TGF-β administration on stiffer substrates.

Previous hydrogel-based models demonstrated their potentialities in the study of cardiac tissue biology and disease progression and in the preclinical testing of drug safety and efficacy. However, the use of mouse/rat cardiac cells limited their relevance, making them poorly predictive of human cardiotoxicity. Indeed, animal-derived cells present differences in molecular mechanisms compared to humans, limiting their translational potential.^22^ Furthermore, one more main limitation of hydrogel substrates is the lack of structural cues needed to reproduce the native cell organization. Additionally, cardiac fibroblasts cultured in hydrogels also need additional biochemical or mechanical stimuli to differentiate into MyoFs.^21^ The exploitation of synthetic polymer scaffolds surface-functionalized with ECM-like polymers could provide the scaffolds proper architectural, chemical, and mechanical cues for human cardiac cell guidance, engineering relevant human cardiac tissue models.

The aim of this work was to design a new *in vitro* model of human cardiac fibrotic tissue mimicking the main characteristics of its early-stage pathological remodeling. Poly(ε-caprolactone) (PCL)-based scaffolds with randomly oriented nanostructures were fabricated by the solution electrospinning technique to provide biomimetic topographical and mechanical cues. Indeed, stiffness and random organization of cardiac fibrotic tissue were reproduced by PCL electrospun scaffolds. Then, the scaffolds were surface-grafted with human type I collagen and fibronectin to mimic the protein microenvironment of the human cardiac fibrotic tissue ECM and to support the culture of human CFs (HCFs), the main cell population of human cardiac fibrotic tissue. A biochemical profibrotic stimulus was integrated in the culture environment by TGF-β addition to the culture medium. Each step of model design was thoroughly characterized by a variety of physicochemical and biological analyses. Immunostaining for α-SMA and quantification of α-SMA-positive cells showed that the model, even in the absence of TGF-β biochemical stimulation, promoted the activation of HCFs into MyoFs. Then, a commercially available antifibrotic drug was tested on the *in vitro* cardiac fibrotic tissue model in order to assess its predictive behavior. Based on the results, the designed *in vitro* human model of early-stage cardiac fibrosis can be exploited as a promising biomimetic platform for the *in vitro* preclinical testing of new advanced regenerative therapies.

## 2 Materials and Methods

### 2.1 Materials

PCL with a weight-average molecular weight of 43,000 g mol^–1^ was purchased from Polysciences. 3,4-Dihydroxy-dl-phenylalanine (DOPA) and human fibronectin were supplied from Sigma-Aldrich, Milano. Human collagen type I was supplied from Corning. All the other reagents were purchased from Sigma-Aldrich.

### 2.2 Electrospun Membrane Preparation

PCL pellets were dissolved in 70% v/v chloroform at room temperature under mild stirring up to complete dissolution. Then, 30% v/v formic acid was added under stirring to obtain a solution with 20% w/v concentration.

The PCL solution was electrospun using a NovaSpider V5 electrospinning equipment (NovaSpider). A syringe with 5 mL capacity was filled and connected through a Teflon tubing to a needle tip placed on a stationary electrospinning stage. The tip-to-collector distance was set at 15 cm. A flat metal collector (190 × 190 mm) and a 22 gauge needle were used. Process parameters were: 15 kV voltage, 0.5 mL/h flow rate, 40–50% humidity and room temperature.

### 2.3 Functionalization of PCL Electrospun Nanofibers

Electrospun PCL membranes with an average thickness of 60 μm were surface-modified by a two-step functionalization process based on a mussel-inspired approach, following the protocol reported by Carmagnola et al.^23^ Briefly, PCL samples were immersed in a slightly basic solution of 3,4-dihydroxy-dl-phenylalanine (2 mg/mL in 10 mM Tris/HCl at pH 8.5) for 7 h at room temperature (PCL/polyDOPA). The solution was kept under 100 rpm stirring rate to enhance DOPA oxidation and self-polymerization. PCL/polyDOPA scaffolds were washed with Tris/HCl buffer solution thrice and, then, rinsed in distilled water. Subsequently, the samples were incubated in a solution of human collagen type I/fibronectin with 70:30 w/w ratio (C1F) in phosphate-buffered saline (PBS; 100 μg/mL) for 16 h at room temperature (PCL/polyDOPA/C1F). The samples were then thoroughly washed with PBS thrice and then with distilled water thrice again to remove unbonded C1/F.

### 2.4 Nanofiber Characterization

#### 2.4.1 Scanning Electron Microscopy

The morphology of the electrospun mats before and after surface modification was analyzed using a scanning electron microscope (FESEM SUPRA 40). PCL and PCL/polyDOPA/C1F samples were coated with a thin gold layer by using an Agar Auto Sputter Coater instrument. The SEM images were taken at different magnifications: 1000×, 2000×, and 5000×. The electrospun nanofiber diameter and pore size were evaluated by analyzing the SEM images (5000×) using ImageJ software. To determine the average fiber size, measurements were conducted in triplicate, taking into account 50 fibers for each image.

#### 2.4.2 Quartz Crystal Microbalance with Dissipation Monitoring (QCM-D) Analysis

QCM-D analysis was carried out to preliminarily evaluate the effectiveness of C1F grafting on the adhesive polyDOPA precoating, using a Q-Sense analyzer (Biolin Scientific) equipped with a gold sensor in static configuration. The deposition of biomimetic C1F coating on polyDOPA was monitored. The temperature was set at 20 °C, and an initialization procedure was performed pipetting 300 μL of Tris/HCl solution on the sensor. After its removal, 300 μL of DOPA solution was pipetted on the sensor and left for 7 h. The solution was removed, and three washes with Tris/HCl were performed. 300 μL of C1F solution was dropped on the polyDOPA-coated gold sensor and left for 16 h. Then, the solution was removed, and the sensor was washed three times with PBS for 5 min and three additional times with distilled water for 5 min. During the test, frequency shift and dissipation were monitored in real time. The protein mass deposited on the sensor, dissipation energy, deposition times, and coating layer thickness were measured using Q-Sense software.

#### 2.4.3 Immunofluorescence

Immunofluorescence analysis was performed on coated PCL/polyDOPA/C1F scaffolds and functionalized gold sensor with the aim to verify the successful deposition of biomimetic coating and the relative distribution/arrangement of type I collagen and fibronectin in the coating. Samples were fixed in paraformaldehyde (PFA, ThermoFisher Scientific) solution (4% v/v) in PBS for 20 min and blocked with bovine serum albumin (BSA, Sigma-Aldrich) solution (1% w/v) in PBS for 30 min. Rabbit anti-fibronectin primary antibody (Sigma-Aldrich) and mouse anti-collagen type I primary antibody (Sigma-Aldrich) solutions were diluted in BSA (1% w/v) in PBS at 1:400 and 1:500, respectively. The samples were incubated in the resulting solutions for 1 h at room temperature and then washed three times in PBS. The anti-rabbit secondary antibody AlexaFluor488 (Life Technologies) and anti-mouse secondary antibody AlexaFluor555 (Life Technologies) were diluted at 1:500 in BSA solution (1% w/v). The samples were incubated in the resulting solutions for 1 h at room temperature and washed three times in PBS. Finally, the samples were imaged under a fluorescence microscope (Nikon Ti2-E) equipped with a digital camera (Nikon Instrument).

#### 2.4.4 C1F Coating Stability Test

Coating stability test was performed using a BCA protein assay kit (Thermo Scientific Pierce), a colorimetric assay for the detection and quantification of grafted protein coating (C1F). PCL/polyDOPA/C1F nanofibers deposited on culture glasses with a diameter of 12 mm were used to perform BCA analysis. BCA test was performed in triplicate after incubating the samples for 0, 1, 2, and 5 days in 2 mL of PBS to quantify the amount of grafted proteins. At each time step, the samples were withdrawn, placed in the wells of a 24-multiwell plate, and treated with 50 μL of PBS and 400 μL of working reagent. After 30 min of incubation at 37 °C, a volume of 100 μL was transferred into a 96-multiwell plate. Absorbance was measured at 562 nm wavelength using a plate reader (Synergy HTX Multi-Mode Microplate Reader, BioTek). Based on the calibration curve, C1F amount was quantified and expressed as protein weight per exposed surface area (μg/cm^2^). The reported values were obtained by subtracting the reading of polyDOPA-coated scaffolds. For each type of scaffold, three samples were analyzed, and data were reported as the average value ± standard deviation.

### 2.5 Scaffolds’ Mechanical Characterization

The mechanical characterization of PCL/polyDOPA/C1F scaffolds was performed by AFM spectroscopy. A spherical indenter was assembled using a tipless cantilever (TL-FM-20 by Nanosensors), and a tungsten sphere of about 10 μm diameter (357421-10G by Aldrich Chemistry) bounded together using an epoxy adhesive cured by UV light. The cantilever spring constant was measured to be 4.77 N/m using thermal noise and the Sader-based method,^24^ while the resonance frequency was about 66.6 kHz and sensitivity was around 38.9 nm/V. Elastic modulus was measured over a grid with 64 points disposed in squares with 20 μm side length; for each point, the measure was repeated at least 10 times.

Hertz’s model over extended curves was applied to measure the local Young’s modulus of scaffolds by the spherical punch model.^25^ Measurements were performed both in air and in water for both scaffold types. The AFM piezo was set to get 150 nN tip force over the sample during the extended segment experimental step. Measurements were performed in triplicate.

### 2.6 Biological Validation

#### 2.6.1 HCF Culture and Seeding on PCL/polyDOPA/C1F Scaffolds

Human cardiac fibroblasts (HCFs) isolated from human ventricle were purchased from PromoCell and maintained in fibroblast growth medium-3 (FGM-3, PromoCell), composed of a basal medium supplemented with 10% fetal calf serum, 1 ng/mL human basic fibroblast growth factor, and 5 μg/mL recombinant human insulin. Cells were maintained at 37 °C in a humidified atmosphere, with 5% CO_2_.

Before cell seeding, PCL/polyDOPA/C1F nanofibers were gently arranged on culture glasses (d: 12 mm) to facilitate handling during culture procedure and analysis. Samples were disinfected by immersion in 70% v/v ethanol (EtOH) for 15 min, followed by rinsing in sterile PBS. The samples were then exposed for 15 min to UV irradiation on each side and finally incubated overnight in 2× antibiotic–antimycotic solution (Life Technologies) in PBS, followed by PBS washes. The scaffolds were seeded with 25,000 cells in a volume of 50 μL. After 1 h incubation, 500 μL of FGM-3 medium was added to each well. The culture medium was replaced every 2–3 days with an equal volume of fresh medium. Cells were then analyzed after 1 and 7 days of culture for cell viability by CellTiter-Blue Cell Viability Assay (Promega) and cytotoxicity by CytoTox-ONE Homogeneous Membrane Integrity Assay (Promega). Cell viability was represented as absolute value of fluorescence at ex/em 560/590 nm, while the cell viability rate was expressed as the percentage ratio between the average cell viability value at 7 d and 24 h, respectively. Control conditions were established by seeding HCFs on gelatin-coated dishes or on tissue culture dish treated with polyDOPA and type I collagen/fibronectin. These analyses were conducted in biological triplicate.

#### 2.6.2 HCF Stimulation with TGF-β

Biochemical profibrotic stimulus was provided with TGF-β addition to the culture medium. Briefly, TGF-β (PeproTech) solution was prepared according to the manufacturer’s instructions and added to the culture medium at a concentration of 2 ng/mL. FGM-3 supplemented with TGF-β was added to PCL/polyDOPA/C1F samples 1 h after cell seeding and replaced every 2/3 days throughout the experiments.

As a proof-of-concept study, HCFs were treated with Tranilast (Sigma-Aldrich), a commercial drug that acts on the TGF-β pathway, previously used in an animal study to counteract cardiac fibrosis.^26,27^ After 24 h of culture on PCL/polyDOPA/C1F samples with/without TGF-β treatment, Tranilast (50 μM) was daily administered to the culture medium until day 7.

#### 2.6.3 Immunofluorescence

Samples were fixed in paraformaldehyde solution (4% v/v) in PBS (PFA, Alfa Aesar) for 15 min, washed with PBS, and cells were permeabilized with Triton X-100 (Sigma-Aldrich) 0.5% v/v in PBS for 10 min. The samples were then blocked with bovine serum albumin (BSA, Sigma-Aldrich) solution (2% w/v) in PBS for 30 min, followed by staining with phalloidin–rhodamine (ThermoFisher) or primary and secondary antibodies, diluted in BSA (2% w/v) in PBS. The primary antibodies for fibroblast staining were: anti-actin smooth muscle (α-SMA, Sigma-Aldrich) and anti-discoidin domain receptor 2 (DDR-2, ThermoFisher). Secondary antibodies used were anti-mouse AlexaFluor555 and anti-rabbit AlexaFluor488 (both from ThermoFisher). Nuclei were counterstained with DAPI (Sigma-Aldrich).

After Tranilast treatment, immunofluorescence quantification for fibronectin, collagen I, and collagen III was performed in order to assess the predictivity of the model for drug efficacy testing. The primary antibodies used for ECM protein detection were anti-fibronectin, anti-collagen I, and anti-collagen III (all purchased from Sigma-Aldrich). Secondary antibodies were anti-mouse AlexaFluor555 and anti-rabbit AlexaFluor488 (both from ThermoFisher). Nuclei were counterstained with DAPI (Sigma-Aldrich). For immunofluorescence signal quantification, five different images for each sample were processed through ImageJ software. After setting the brightness/contrast/threshold and creating a ROI on the binary image, the percentage area for a single-color channel was estimated. For each analyzed image, the estimated fluorescence intensity was normalized with respect to the number of cell nuclei.

Samples were maintained in PBS during imaging by using a Nikon Ti2-E fluorescence microscope (Nikon Instruments). Immunofluorescence experiments were performed in biological triplicates.

### 2.7 Statistical Analysis

All experiments were performed in triplicates, and data were presented as mean ± SD from three independent experiments. Data were analyzed with GraphPad Prism version 9.0 for Windows (GraphPad Software, www.graphpad.com), using one-way ANOVA analysis to compare the results.

## 3 Results

The aim of this work was to engineer an *in vitro* model mimicking the main features of human post-infarct fibrotic tissue. Native pathological cardiac tissue is characterized by random tissue architecture, stiffening, ECM remodeling with collagen overdeposition, significant increase in fibronectin expression, activation of cardiac fibroblasts into MyoFs, and release of profibrotic factors and cytokines (such as TGF-β). Previous *in vitro* models of pathological cardiac tissue have been engineered by culturing cardiac fibroblasts into hydrogel substrates, which however lack architectural cues able to drive cell organization.^19,20^ Furthermore, the formation of MyoFs has been mainly triggered by biochemical factors, such as the administration of TGF-β, or by cyclic mechanical stimulation (alone or combined with TGF-β release),^28^ while biomimetic scaffolds with the intrinsic ability to support MyoF formation and culture are missing.

Electrospun scaffolds based on PCL, surface-functionalized with pathological cardiac ECM mimetic molecules, were herein designed and cultured with HCFs from the cardiac ventricle. The effects of biomimetic scaffold morphology, surface chemical composition, and stiffness on HCF arrangement, proliferation, and activation were analyzed, showing the scaffold ability to support the design of *in vitro* models of early-stage cardiac fibrotic tissue.

### 3.1 Electrospun Scaffolds’ Morphological Characterization

**Figure 1:**
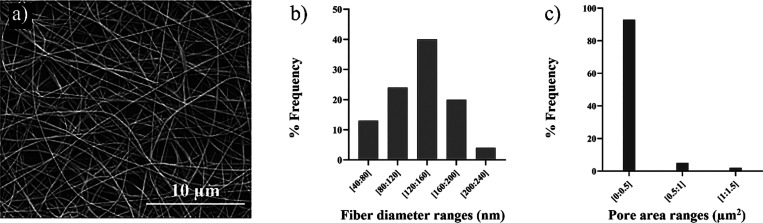
SEM image of the PCL electrospun nanofibrous membrane (a); percentage distribution of fiber size (b); and percentage distribution of pore area (c).

The morphology of electrospun PCL membranes was analyzed by SEM to select the optimal process parameters, leading to the formation of randomly oriented nanofibers free of defects. Electrospun PCL membranes showed a nanofibrous morphology (Figure 1a) with an average thickness of around 60 μm. The geometrical features of membranes were derived by SEM images using ImageJ software. The percentage distributions of the fiber diameter and pore area are reported in Figure 1b,c. The fibers showed an average diameter of 131 ± 39 nm, whereas pores showed an average area below 0.5 μm^2^ (Figure 1c).

### 3.2 C1F Grafting Validation

#### 3.2.1 QCM-D Analysis

Collagen type I and fibronectin were chosen as the two main components of cardiac pathological ECM to functionalize PCL electrospun membranes, obtaining biomimetic “bioartificial” scaffolds. C1F blend composition was selected based on the reported literature data on the pathological cardiac ECM composition.^13,29,30^ PCL electrospun membranes were prefunctionalized with polyDOPA, following a previously optimized protocol^23^ and, then, grafted with C1F and single molecules (type I collagen and fibronectin) as controls.

**Figure 2:**
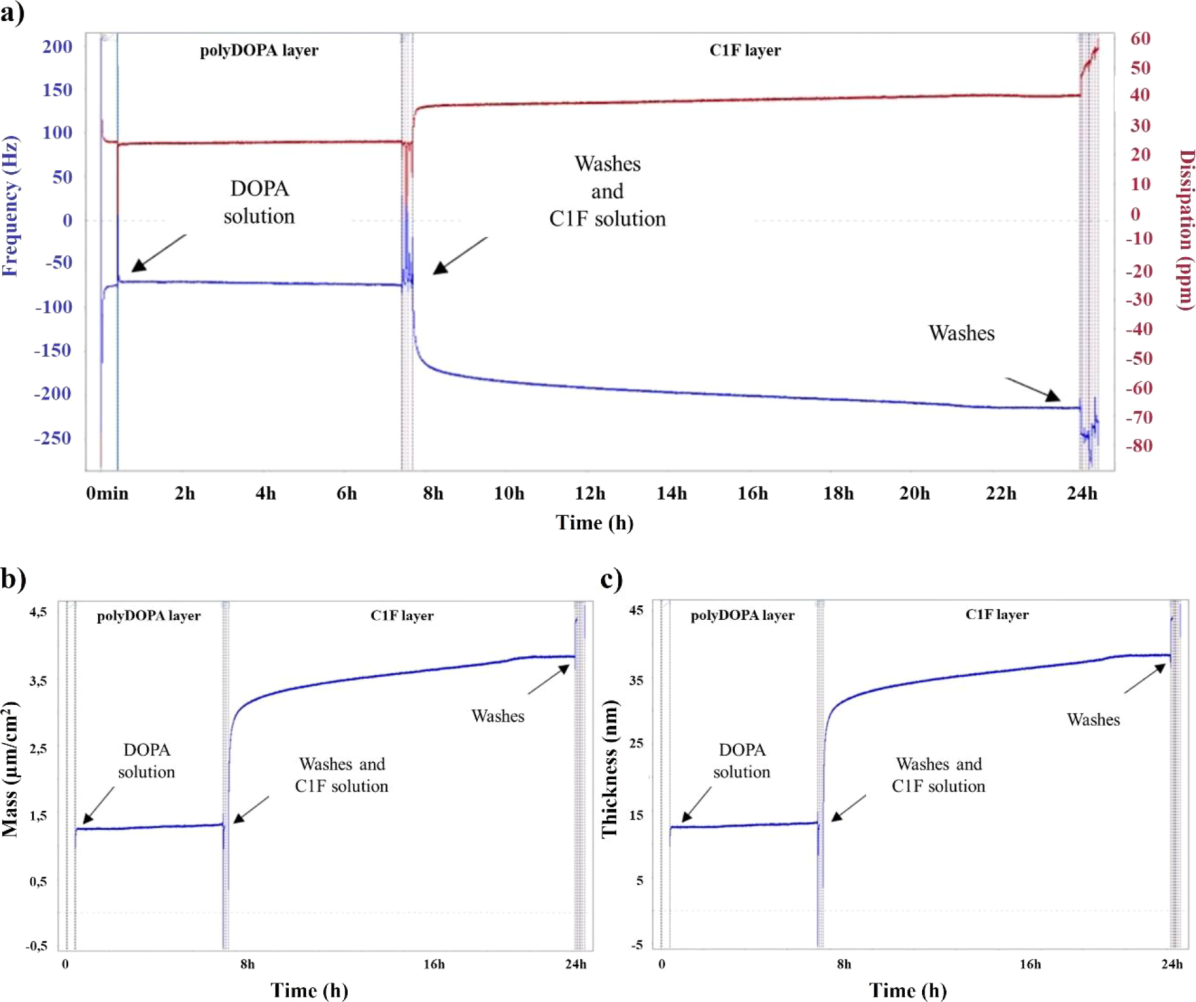
(a) Behavior of frequency shift expressed in Hz (blue) and dissipation expressed in ppm (red) as a function of time during the deposition of polyDOPA (first 7 h) and C1F (subsequent 16 h); (b) mass and (c) thickness of polyDOPA and C1F layers as a function of time, estimated through “Sauerbrey” and “Smartfit” models, respectively.

The protein grafting efficiency on polyDOPA coating was preliminarily assessed by QCM-D analysis on a polyDOPA-coated sensor. Frequency shifts and energy dissipations were monitored in real time and reported as a function of functionalization time in Figure 2a for C1F and in Figures S1a and S2a for type I collagen and fibronectin, respectively. The successful deposition of the polyDOPA layer was proven by the detected frequency shift between 20 and 100 Hz in the three experimental conditions. After 7 h of incubation with the DOPA solution, the frequency and dissipation signals appeared noisy due to sensor washing and subsequent addition of type I collagen, fibronectin, or C1F solutions (Figures S1a, S2a, and 2a). The addition of protein solutions caused a sudden decrease in frequency shift and an increase in dissipation; both trends were preserved over the next 16 h of incubation in protein solutions. Successful C1F grafting was demonstrated by the frequency decrease from around −70 to about −296 Hz after 16 h of incubation (Figure 2a).

The mass and thickness of C1F (Figure 2b,c) and control coatings (Figures S1b,c and S2b,c) were estimated from the frequency values at the end of each deposition step. The values of coating mass and thickness in the three experimental conditions are summarized in Table S1. At the end of the C1F grafting process, the overall mass deposited per unit area was 5.3 μg/cm^2^. Based on the sensor surface area (1.54 cm^2^), layer mass was calculated. As reported in Table S1, the overall quantity of the deposited C1F was 5.5 μg, confirming the successful deposition of the biomimetic coating on adhesive polyDOPA. Concerning layer thickness, the polyDOPA layer was 17.2 nm thick, and C1F coating reached a thickness of 35.8 nm (Figure 2c and Table S1).

#### 3.2.2 Immunofluorescence Staining

**Figure 3:**
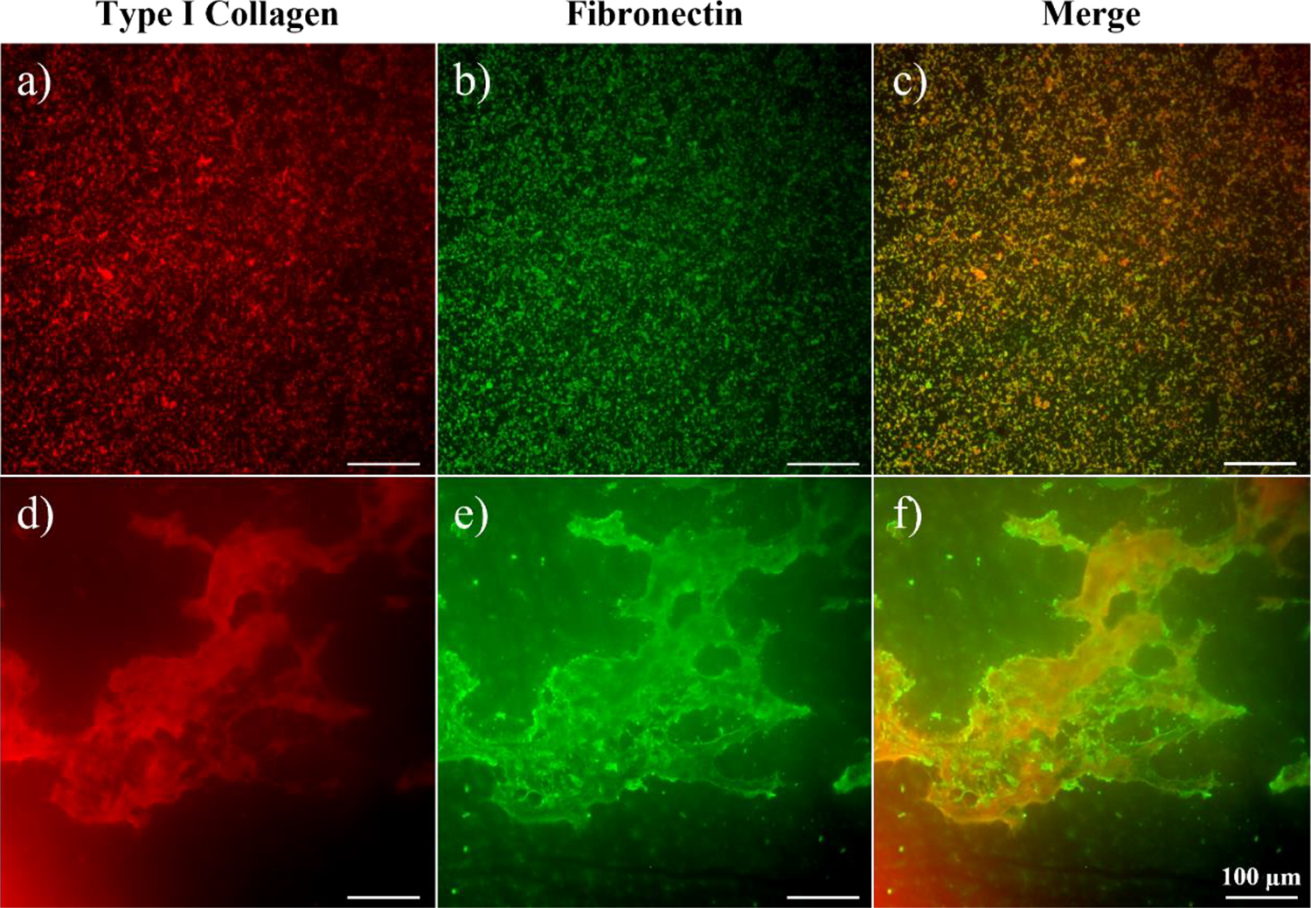
Immunofluorescence analysis of type I collagen (red) (a, d), fibronectin (green) (b, e), and their merge (c, f) on C1F-grafted polyDOPA-coated gold sensor (a– c) and the nanofibrous membranes (d– f). Scale bar is 100 μm.

C1F-functionalized QCM sensors were analyzed by immunofluorescence analysis, confirming the successful grafting of type I collagen and fibronectin and characterizing their surface distribution (Figure 3).

Immunofluorescence images confirmed the successful grafting of collagen type I and fibronectin on a polyDOPA-coated sensor (Figure 3a–c). Interestingly, a punctiform distribution of both proteins was detected, showing a homogeneous fluorescence intensity distribution, suggesting a uniform spatial distribution of the proteins in the coating. In several regions of the sensor, the two fluorescence signals overlapped, suggesting the co-presence of both proteins (Figure 3c).

### 3.3 Electrospun Scaffolds’ Surface Functionalization

#### 3.3.1 Immunofluorescence Staining

The surface functionalization protocol validated through QCM-D analysis was then applied to PCL electrospun membranes. In order to characterize coating formation and protein distribution, immunofluorescence analysis was performed on C1F-grafted polyDOPA-coated PCL membranes (Figure 3d–f). Figure 3d,e shows that the spatial distribution and arrangement of the two proteins (fibronectin in green and type I collagen in red) on the polyDOPA-coated PCL membrane surface was significantly different than that on polyDOPA-coated gold sensors. Guided by the PCL nanofibrous structure, the proteins assembled each other rather than assuming a punctiform morphology as on the polyDOPA-coated QCM sensor. Such protein arrangement was attributed to reciprocal fibrillogenesis of collagen type I and fibronectin, also suggested by wide fluorescence signal overlapping (Figure 3f).^31^

#### 3.3.2 BCA Protein Assay

**Figure 4:**
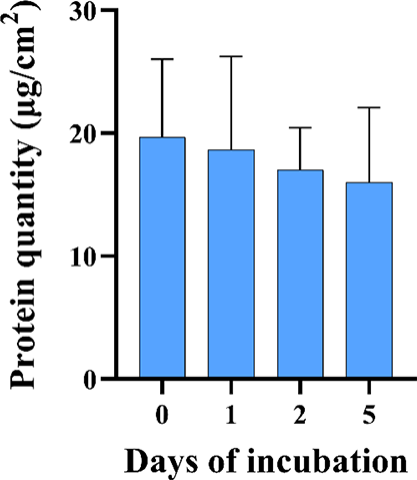
C1F amount analyzed by BCA assay at 0, 1, 2, and 5 days of incubation in PBS at 37 °C. Grafted protein density was reported as μg/cm^2^.

BCA colorimetric assay was carried out to quantify the amount of grafted C1F onto PCL/polyDOPA electrospun membranes on as-prepared samples and after their incubation in PBS at 37 °C (Figure 4). The average amount of grafted protein on polyDOPA-coated samples at t0 was 20 μg/cm^2^ per exposed surface of the electrospun scaffold. The histogram in Figure 4 shows a decreasing trend of the average density of grafted proteins as a function of incubation time in PBS but with no statistically significant differences. Therefore, in wet conditions, coating was stable up to 5 days.

### 3.4 Surface Mechanical Characterization by AFM Analysis

**Figure 5:**
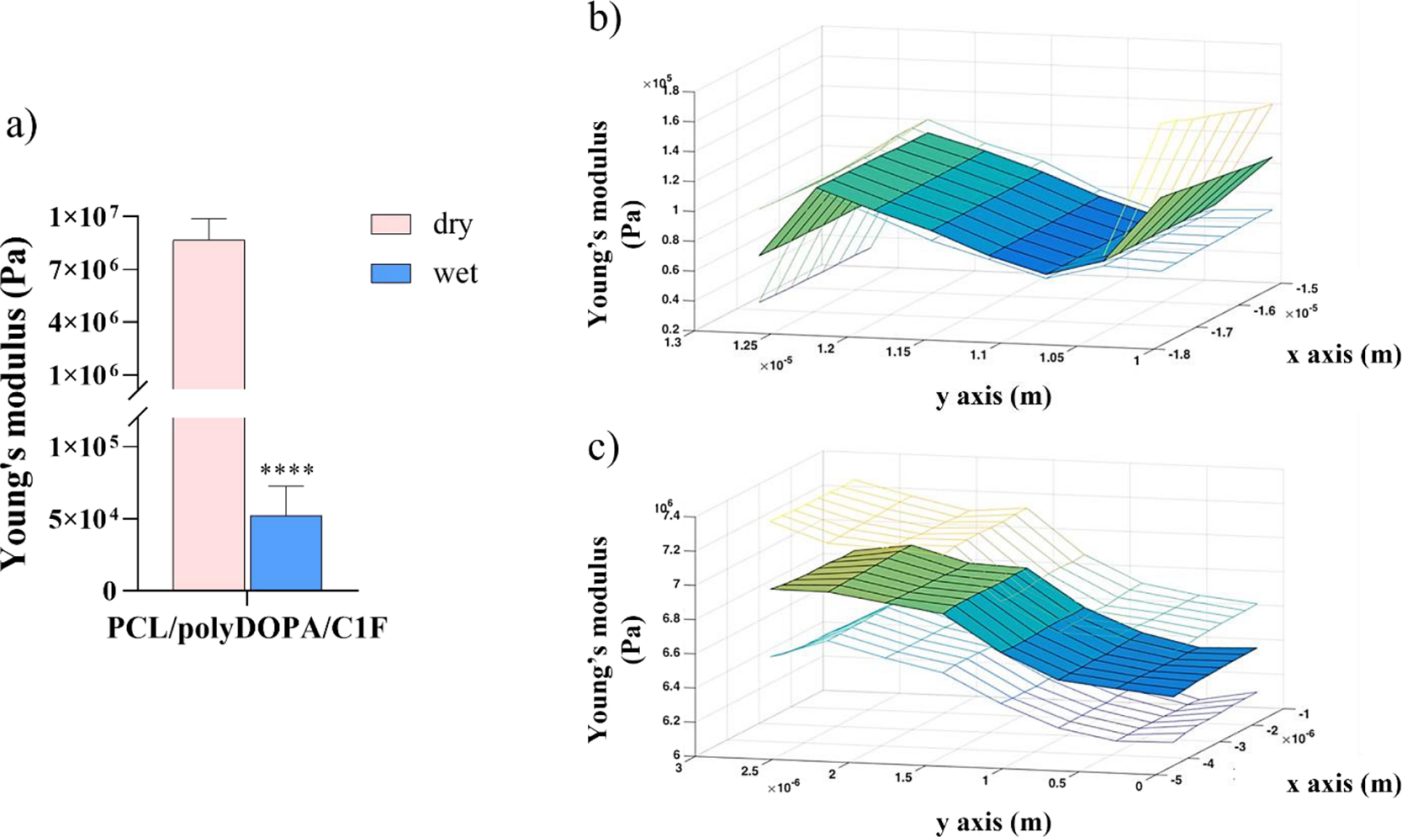
Young’s modulus measured by AFM on PCL/polyDOPA/C1F membranes in both dry and wet conditions: average Young’s modulus values (a); surface distribution of Young’s modulus values measured in dry (b) and wet conditions (c) ****p < 0.0001.

AFM was performed to characterize the mechanical behavior of electrospun membranes before and after the surface modification process. In detail, PCL and PCL/polyDOPA/C1F samples were tested in dry and wet conditions. The histogram in Figure 5a shows Young’s modulus values for PCL/polyDOPA/C1F samples in the two experimental conditions, while data for PCL samples are reported in Figure S3a. For PCL samples (Figure S3a), the elastic modulus increase is around 2 orders of magnitude from about 9 × 10^4^ Pa in dry conditions to 1 × 10^6^ Pa in wet conditions, suggesting the stiffening of PCL nanofibers in the wet environment.

For PCL/polyDOPA/C1F samples (Figure 5a), the trend of Young’s modulus as a function of experimental conditions was opposite compared to PCL samples: the average value of the Young’s modulus decreased significantly from 8.7 × 10^6^ Pa in dry state to 5.2 × 10^4^ Pa in wet conditions.

Figure 5b,c shows the spatial distributions of Young’s modulus in the *XY* plane of indentation in dry and wet conditions, respectively.

Colormaps reflect Young’s modulus values for each point of the experimental grid in wet and dry conditions for PCL (Figure S3b,c) and PCL/polyDOPA/C1F membranes (Figure 5b,c).

### 3.5 HCF Viability and Cytotoxicity

**Figure 6:**
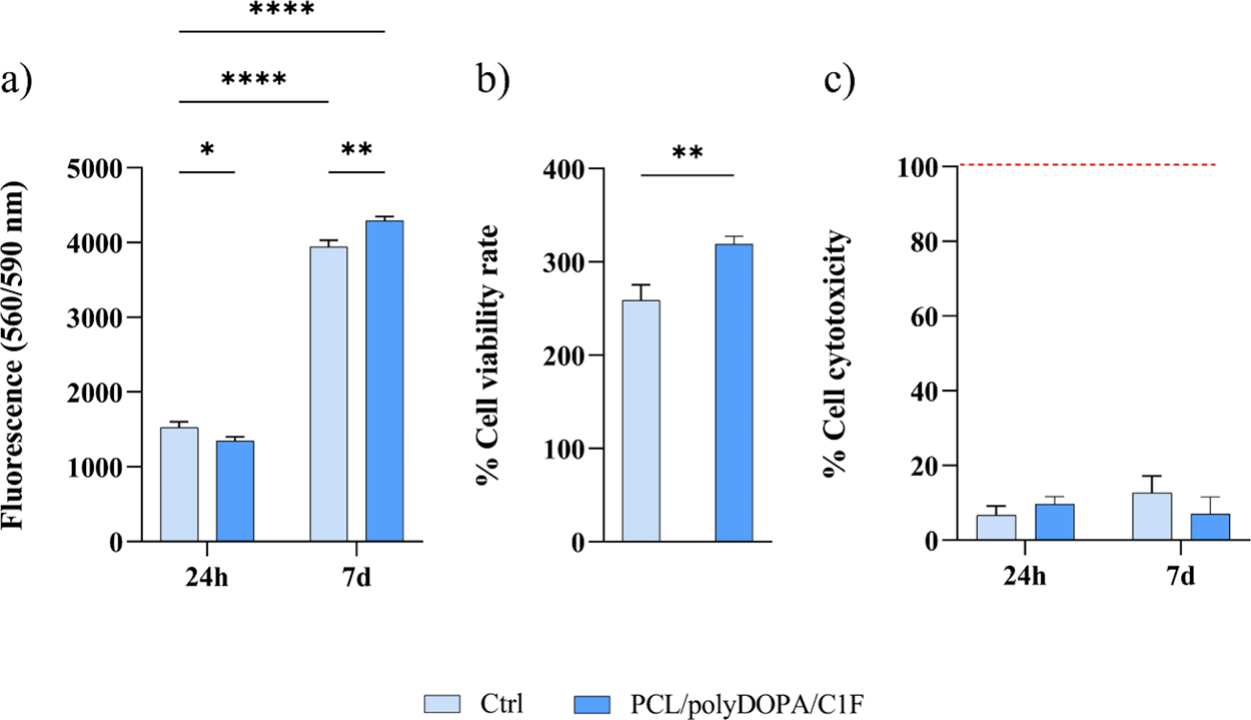
Fluorescence intensity (560/590 nm) values by resazurin cell viability assay after 24 h and 7 days of culture (a). Percentage of cell viability rate at 7 days over 24 h culture time (b). Cytotoxicity percentage after 24 h and 7 days culture time, referred to 100% cell lysis control consisting of cells cultured on gelatin-coated glass and treated with lysis buffer (9% Triton X-100 in water) (c). Data for control samples (Ctrl) are shown in light blue, while results obtained by cell culture on PCL/polyDOPA/C1F scaffolds are shown in dark blue (*p < 0.05, **** *p* < 0.0001).

Cytocompatibility and cytotoxicity of HCFs cultured on PCL/polyDOPA/C1F membranes and gelatin-coated dishes (control) for 24 h and 7 days were evaluated. As shown in Figure 6a, the HCF viability was slightly lower on PCL/polyDOPA/C1F membranes than on control conditions at 24 h, while after 7 days, the cell viability on PCL/polyDOPA/C1F membranes became significantly higher. The increase of cell viability suggested a higher number of cells metabolizing resazurin. The percentage of cell viability rate at 7 d over 24 h for control samples and PCL/polyDOPA/C1F membranes was also reported (Figure 6b), showing values of ∼260% and 320%, respectively. Cytotoxicity data (Figure 6c) indicated no cytotoxic effects on cells (<20% cytotoxicity for all conditions), with no significant differences between cells on control samples and PCL/polyDOPA/C1F membranes.

### 3.6 HCF Phenotype Characterization and TGF-β Mediated Activation

**Figure 7:**
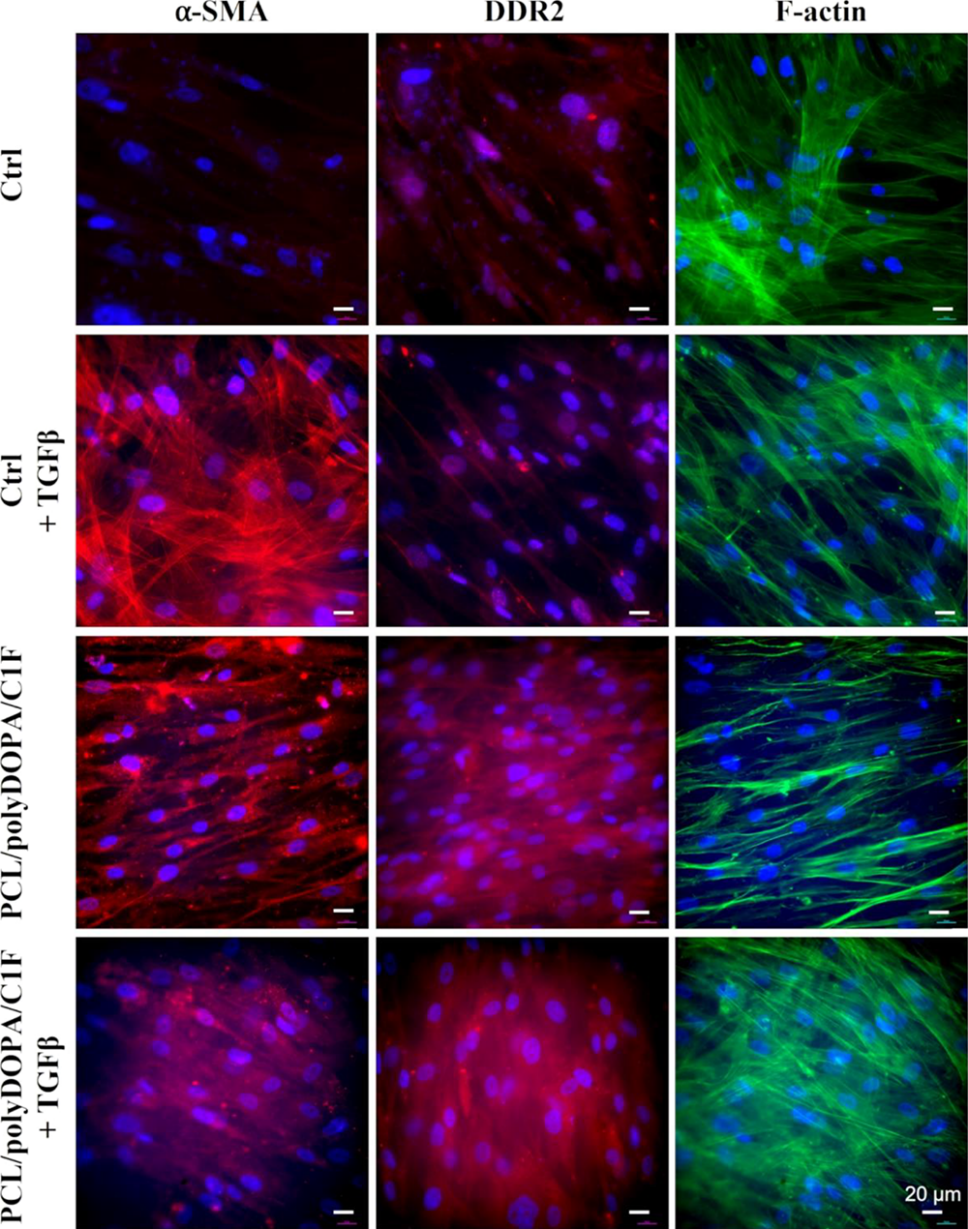
Immunostaining for α-SMA (red), DDR2 (red), and F-actin (green) on HCFs cultured for 7 days on gelatin-coated dishes (control samples, Ctrl) and PCL/polyDOPA/C1F membranes, with and without TGF-β addition (2 ng/mL). Nuclei (blue) were counterstained with DAPI.

**Figure 8:**
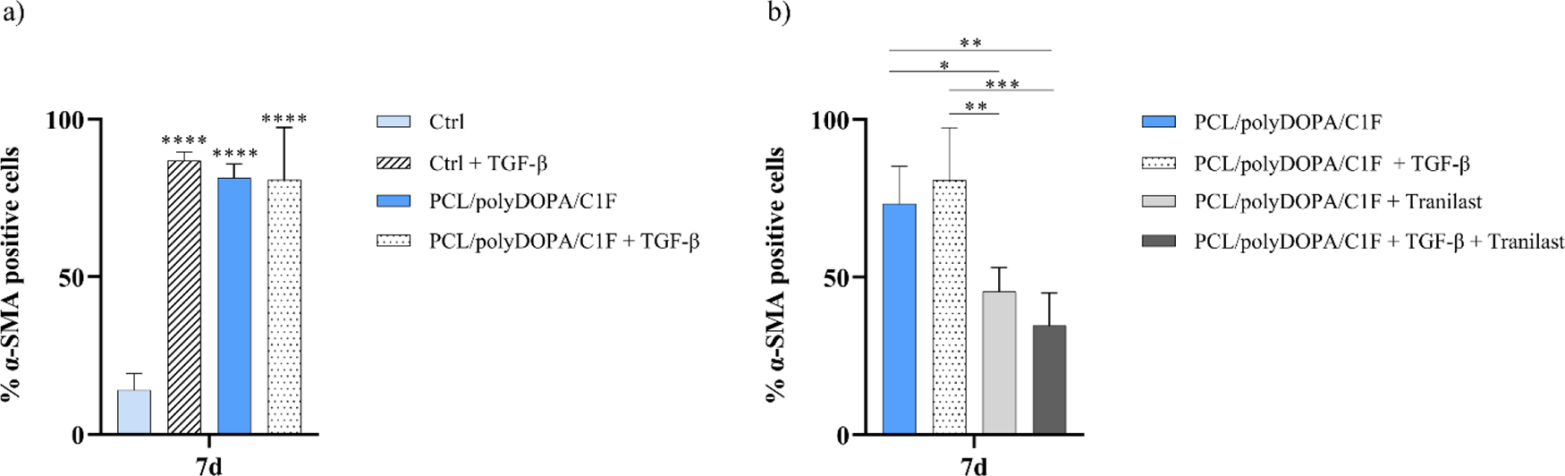
(a) Percentage of α-SMA-positive cells over the total nuclei, counted after 7 days of culture on control samples (Ctrl) and PCL/polyDOPA/C1F membranes with and without TGF-β stimulation. Significance is referred to Ctrl condition. (b) Percentage of α-SMA-positive cells over the total nuclei with and without Tranilast treatment, counted after 7 days of culture on PCL/polyDOPA/C1F membranes with and without TGF-β stimulation (*p < 0.05, ****p < 0.0001).

After an ischemic event, the release of TGF-β activates HCFs into MyoFs, which is characterized by the overexpression of the cytoskeletal contractile protein α-SMA. The effects on the cell phenotype of HCF culture on PCL/polyDOPA/C1F membranes with/without TGF-β stimulation (2 ng/mL) were evaluated over 7 days. The expressions of both α-SMA and DDR2 protein, the typical receptors expressed by cardiac fibroblasts and binding collagen, were evaluated (Figure 7). For all conditions, F-actin staining suggested an adequate cell coverage on all culture substrates and control conditions (gelatin-coated Petri dishes). HCFs cultured in PCL/polyDOPA/C1F membranes showed high expressions of both α-SMA and DDR2, suggesting HCF activation and preservation of the cardiac phenotype with and without TGF-β stimulation. On the contrary, under control conditions, DDR2 was not expressed (with and without TGF-β), while α-SMA was only expressed in the presence of TGF-β. Following the obtained results, the contribution of PCL/polyDOPA/C1F membranes on HCF activation was analyzed. Figure 8 shows the percentage of α-SMA-positive HCFs over total nuclei under control conditions and PCL/polyDOPA/C1F membranes with and without TGF-β stimulation.

Results showed an activation of approximately 80% of HCFs cultured on PCL/polyDOPA/C1F membranes, both with and without TGF-β stimulation, compared to the activation of less than 20% of HCFs on control condition with TGF-β stimulation. Hence, *in vitro* HCF culture on PCL/polyDOPA/C1F scaffolds with biomimetic architectural, chemical, and mechanical properties was found to promote the formation of MyoFs, without the need for additional biochemical stimulation with TGF-β.

### 3.7 Model Validation: Tranilast Effect on Activation and ECM Deposition

**Figure 9:**
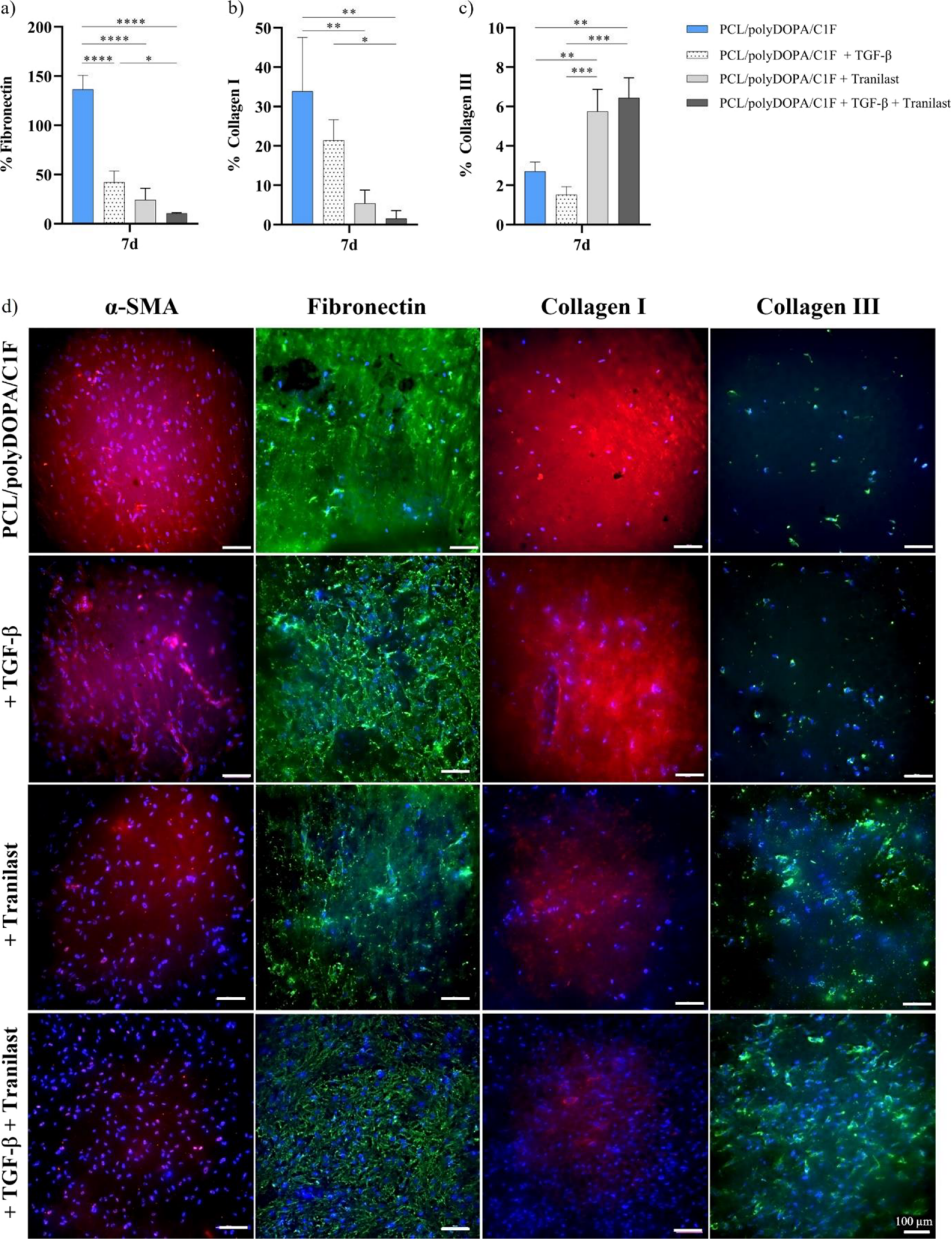
Quantification of immunofluorescence intensity signal from confocal images, as ECM protein percentage over the total nuclei at 7 days’ culture on PCL/polyDOPA/C1F membranes, with/without TGF-β and with/without Tranilast treatment: (a) fibronectin, (b) collagen I, (c) collagen III (*p < 0.05, ****p < 0.0001). (d) Immunostaining for α-SMA (red), fibronectin (green), collagen I (red), and collagen III (green) on HCFs cultured for 7 days on PCL/polyDOPA/C1F membranes, with/without TGF-β and with/without Tranilast treatment; nuclei (blue) were counterstained with DAPI.

The predictivity of the model as a tool for the *in vitro* preclinical assessment of drug efficacy was demonstrated by the administration of Tranilast, a commercially available antifibrotic drug. Tranilast was administered after 24 h culture of HCFs on PCL/polyDOPA/C1F, treated or untreated with TGF-β, followed by *in vitro* culture up to 7 days. The antifibrotic efficacy of Tranilast was evaluated by analyzing the expression of α-SMA myofibroblast marker (Figure 8b) and the deposition of ECM protein (fibronectin, collagen I, and collagen III, Figure 9a–c) by the treated cells. Figure 8b shows that, independent of TFG-β stimulus, Tranilast treatment significantly decreased or prevented HCF activation into myofibroblasts, as evidenced by a remarkable decrease in α-SMA-positive cell percentage over total nuclei (compared to Tranilast untreated cells).

Immunofluorescence quantification of ECM proteins (Figure 9a–c) shows that Tranilast significantly reduced the secretion of fibronectin (Figure 9a) from about 135 to 25% and from about 42 to 10%, for models prepared without and with TGF-β, respectively. A similar decreasing trend was observed for type I collagen (from around 33 to 5% and from about 21 to 1.5% for models prepared without and with TGF-β, respectively. On the opposite, the percentage of type III collagen significantly increased after Tranilast administration (Figure 9b,c) from about 3 to 6% for models prepared without and with TGF-β, respectively. Figure 9d shows the representative merge images (α-SMA/DAPI, fibronectin/DAPI, collagen I/DAPI, collagen III/DAPI) from confocal microscopy, referring to previously mentioned immunofluorescence quantifications.

## 4 Discussion

Myocardial fibrosis consists of the cardiac interstitium expansion due to the dense accumulation of ECM proteins, and it is responsible for most cardiac chronic conditions.^5,10^ During tissue remodeling, activated fibroblasts (MyoFs) are the central effectors of fibrosis since they are ECM-secretory cells, acting as the main source of structural ECM proteins.^10^ The overdeposited fibrotic ECM appears rich in collagens (mainly type I), fibronectin, laminin, and elastin.^29,30,32^ Particularly, the increased expression of type I collagen and the accumulation of type III fibrillar collagen are well-known hallmarks of cardiac fibrosis since they directly compromise the structural integrity of the cardiac tissue, altering its biochemical, mechanical, and electrical coupling properties. More in detail, the deposition of a collagen-based matrix, related to the persistent activation of MyoFs, establishes the end of the proliferative phase and the beginning of scar maturation, characterized by protein cross-linking. The dense cross-linked collagen matrix not only provides a strong structural protection for post-MI heart tissue but also enhances tissue stiffness, contributing to diastolic dysfunction and favoring the occurrence of arrhythmias.^33,34^

The aim of *in vitro* engineering of human fibrotic tissue models is to provide predictive platforms with relevant cell types and biomimetic microenvironment, allowing an *in vitro* preclinical assessment of new advanced regenerative strategies or to explore the pathogenesis of cardiac fibrosis.^35^ In the field of cardiovascular diseases, during the last decades, the use of 2D cultures has been exploited to understand myocardial fibrosis mechanisms. However, 2D cultures do not recapitulate the *in vivo* tissue complexity, despite promoting the activation of MyoFs at certain extents. A few *in vitro* models of cardiac fibrosis have been previously proposed; however, they have shown poor predictivity of human pathophysiology as mainly based on cardiac cells derived from neonatal mouse or rat hearts.^19,21,36^ Furthermore, hydrogel substrates have been mainly used for pathological cardiac tissue modeling, but they are unable to provide architectural cues to cells and need additional biochemical stimulation for fibroblast activation into MyoFs.

In this work, we designed a scaffold-based model combining biomimetic topographical, mechanical, and chemical cues to *in vitro* engineer human cardiac fibrosis, overcoming the limitations of the previously developed models. HCFs were cultured and stimulated on scaffolds mimicking *in vivo-*like post-infarct microenvironment.^13,37^ First, ECM-like nanofibrous substrates mimicking pathological cardiac tissue were prepared. PCL-based membranes with randomly oriented nanofibrous morphology were successfully fabricated by solution electrospinning. SEM analysis (Figure 1a) showed that PCL membranes were free of defects, containing randomly oriented nanofibers with homogeneous morphology, with an average size of 131 ± 39 nm (Figure 1b) and uniformly distributed pore area within 0–0.5 μm^2^ range (Figure 1c).

In heart remodeling, as well as fibrillar collagen synthesis, ECM is subjected to dynamic compositional changes in order to support the proliferation, migration, and activation of MyoFs phenotype. During the early stage of fibrotic response, a temporary matrix network results from the extravasation of plasma proteins (mainly fibronectin), due to increased microvascular permeability.^10,38^ This dynamic network is directly implicated in the regulation of crucial cellular processes like fibroblast proliferation and activation. In this work, in order to replicate the ECM post-infarct composition in terms of key proteins involved in fibrosis, PCL nanofibrous membranes were surface-grafted with human type I collagen and fibronectin (C1F coating) with 70/30 w/w ratio. This composition was defined according to previous studies on pathological cardiac ECM composition.^13,39,40^ In a recent study, we obtained a pathological cardiac ECM coating on PCL scaffolds by a different approach based on culturing HCFs for 3 weeks on scaffolds, followed by decellularization.^41^ Although ensuring relevant biomimicry, such an approach was complex and time-consuming. Furthermore, in the absence of any additional stimulation, HCFs did not deposit an adequate amount of type I collagen on scaffolds after 3 weeks’ culture. The advantages of the new coating approach are related to its superior reproducibility and biomimicry thanks to the use of a protein blend, containing type I collagen. C1F coating was grafted on PCL scaffolds after the deposition of an adhesive layer of polyDOPA, following a previously optimized protocol.^23^

The effectiveness of C1F grafting on the polyDOPA precoating was preliminarily assessed by QCM-D on the polyDOPA-coated gold sensor (Figure 2). Coating with type I collagen and fibronectin alone on the polyDOPA precoating was also studied to establish the optimal treatment time for successful protein grafting. Frequency shift and energy dissipation were monitored in real time and reported as a function of incubation time (Figures S1a and S2a). Protein grafting caused an initial slight frequency decrease, suggesting protein deposition at the sensor interface. Specifically, for sensor samples coated with type I collagen, fibronectin, and C1F, a plateau in frequency shift was reached after 10, 6, and 14 h of incubation, respectively. C1F grafting (Figure 2a) was demonstrated by the frequency decrease of around 220 Hz after overnight incubation. In order to ensure high grafting efficiency for both proteins, 14 h incubation was selected for subsequent C1F coating of PCL/polyDOPA membranes. Besides, depending on the coating layer stiffness, different models were applied to estimate the mass change by the measured dissipation. Particularly, “Sauerbrey” and “Smartfit” models were found to be appropriate to estimate polyDOPA and C1F coating mass and thickness, respectively.^42^ Interestingly, the total amount of deposited C1F (Table S1) was higher than that resulting from the simple additive rule. The high reciprocal affinity of type I collagen and fibronectin probably enhanced the amount of proteins grafted on the membranes. Indeed, fibronectin was previously found to accelerate the early stages of collagen assembly, while collagen fibrillogenesis was found to promote the formation of fibronectin fibrils.^31^ Immunofluorescence images of polyDOPA/C1F-coated sensor confirmed the homogeneous deposition of C1F (Figure 3a–c), although assembled in a nonbiomimetic spot-like arrangement. On the other hand, immunofluorescence analysis of PCL/polyDOPA/C1F membranes confirmed the deposition of C1F with an ECM-like assembly on scaffolds, probably supported by the nanostructured topography of membranes lying beneath (Figure 3d–f). C1F coating composition and arrangement were responsible for the chemical biomimicry of PCL/polyDOPA/C1F membranes to human cardiac fibrotic ECM. Interestingly, C1F coating showed stability after 5 days of incubation in PBS solution (Figure 5), due to its covalent grafting to the polyDOPA layer.^23^ The stable C1F coating promoted HCF adhesion, proliferation, and differentiation, as demonstrated by subsequent *in vitro* cell tests. HCF behavior is regulated by several mechanobiological cues in the native microenvironment, as suggested by *in vitro* and *in vivo* HCF response to increased ECM stiffness or cyclic mechanical strains, undergoing differentiation into MyoFs.^29,44^ Previous studies on ECM dynamics demonstrated that ECM regulates fibrosis both chemically and mechanically. The transformation of mechanical signals into biochemical signals plays a pivotal role in cell differentiation.^29,43,44^ Particularly, human fibrotic cardiac tissue is hallmarked by an increased mechanical stiffness (∼30–100 kPa) compared to healthy myocardial tissue (∼10 kPa).^21^ For this reason, PCL/polyDOPA/C1F membranes were mechanically characterized by AFM in wet and dry conditions. Substrate mechanical stiffness was investigated in wet conditions in order to estimate the mechanical behavior in an *in vitro-*like culture environment (Figure 5). For PCL membranes, the average value of Young’s modulus increased from about 9 × 10^4^ Pa in dry condition to 1 × 10^6^ Pa in wet condition (Figure S3a). As PCL is weakly hydrophilic, PCL nanostructured membranes were hydrophobic (Figure S3a). Consequently, poor affinity with water might explain the enhanced stiffness of PCL membranes in wet state. On the other hand, the presence of C1F coating strongly decreased Young’s modulus of nanostructured PCL/polyDOPA/C1F membranes in wet versus dry conditions. Coated membranes were hydrophilic, and water was absorbed by the natural polymer coating. The average value of Young’s modulus for PCL/polyDOPA/C1F membranes decreased from 8.7 × 10^6^ Pa in dry condition to 5.2 × 10^4^ Pa in wet state (Figure 5a). The latter value was in the range of reported stiffness values for cardiac fibrotic tissue, suggesting the high mechanical biomimicry of polyDOPA/C1F/PCL membranes. Colormaps in Figure 5b,c show the spatial distributions of Young’s modulus in the *XY* plane of indentation on polyDOPA/C1F/PCL scaffolds in wet and dry conditions. The color distribution and the variable height of peaks at the points of investigation demonstrated the nonmechanical uniformity of PCL substrates, due to their randomly oriented nanofibrous structure, characterized by random fiber junctions and pore distribution affecting indentation measures (Figure S3b,c). In the case of PCL/polyDOPA/C1F membranes (Figure 5b,c), C1F coating was able to decrease the variability of Young’s modulus values.

As previously mentioned, fibrotic heart is mainly populated by cardiac fibroblasts that, in the presence of the multiple biochemical and biophysical stimuli characterizing the cardiac pathological tissue, differentiate into MyoFs, followed by enhanced secretion of ECM proteins.

To understand the effect of different environmental stimuli on HCF phenotype activation, PCL/polyDOPA/C1F membranes were cultured with HCFs with/without TGF-β stimulation. Substrates supported higher cell viability rate compared to control conditions (Figure 6b), indicating that C1F coating stimulated HCF proliferation. This result was in agreement with previous studies, showing that the presence of type I collagen exerts an enhancing effect on cardiac fibroblast proliferation.^45^ HCFs cultured on PCL/polyDOPA/C1F membranes also showed an enhanced expression of DDR2, a collagen-activated receptor, that has been found to play a pivotal role in promoting cell proliferation, in different pathological conditions associated with exacerbated ECM deposition, such as skin wound healing, liver fibrosis, and even tumor progression.^46^

Moreover, PCL/polyDOPA/C1F membranes triggered HCF activation into MyoFs in 7 days, compared to the control culture conditions, as shown in Figure 8. The addition of TGF-β did not significantly change the percentage of MyoFs on PCL/polyDOPA/C1F membranes, while it exerted a moderate effect on HCFs cultured in control conditions, triggering the activation of 80% MyoFs compared to 20% without stimulus (Figure 8). Usually, *in vitro* conversion of HCFs into MyoFs has been driven by biochemical stimulation through TGF-β addition or by cyclic mechanical strain.^47^ However, in our experiments, HCFs changed their phenotype into MyoFs by the sole effect of the culture substrate, attributed to the biomimetic scaffold architecture and surface stiffness and the presence of C1F coating mimicking fibrotic ECM microenvironment.^48^ The influence of C1F coating, biomimetic surface stiffness, and topography on the activation of HCF signaling pathways would need deeper investigations which are out of the scope of this work.

Finally, the ability of the model to recapitulate human cardiac fibrotic tissue response upon antifibrotic drug administration was analyzed by its treatment with the commercially available drug Tranilast. Tranilast is a fattening cell stabilizer, mainly used to treat allergic reactions and has already been shown to inhibit TGF-β-related fibrosis and organ failure in various disease models, including post-MI myocardial fibrosis.^26,27^ Furthermore, the administration of exogenous TGF-β has been found to stimulate TGF-β gene expression in cultured cardiac fibroblasts. This effect is dose-dependent and prevented by the addition of Tranilast to the culture medium.^26,27,49^ The decrease of α-SMA expression (Figure 8b) and the reduced amount of fibronectin and collagen I (Figure 9) demonstrated Tranilast efficacy and, consequently, model reliability and predictivity.

Overall, the developed model was demonstrated to recapitulate the main adverse tissue remodeling effects occurring post-MI. However, the model was simplified and only included cardiac fibroblasts, being the main cell types contributing to the formation of cardiac scar tissue, starting from early inflammatory phase and proceeding through proliferative and maturation stages. Full understanding of the pathophysiology mechanism leading to cardiac fibrotic tissue formation would require a closer mimic of the *in vivo* complexity, with the inclusion of other relevant cell types. Indeed, crosstalk of cardiac fibroblasts with other cell types, such as macrophages, cardiomyocytes, and endothelial cells, is fundamental in the regulation of fibroblast phenotype and the reparative process. Therefore, co-culture of cardiac fibroblasts with other cell types could further increase the physiological relevance of the model and allow a more close reproduction of specific stages occurring in the cardiac reparative process. Additionally, the developed model accurately mimics the architectural, compositional, and mechanical features of post-MI fibrotic cardiac tissue with limited thickness, while it is unable to reproduce 3D fibrotic cardiac tissue.

Under profibrotic stimulation, the levels of proinflammatory growth factors and cytokines increase and, then, trigger the activation of signaling pathways and transcriptional factors via Smad-dependent or Smad-independent ways, reinforcing the fibrotic response.^50^ We demonstrated that the administration of TGF-β, the mostly used profibrotic growth factor, was unable to affect the *in vitro* fibrotic response of HCFs cultured on PCL/polyDOPA/C1F scaffolds (Figures 7 and 8a). In the future, the integration in the model of other physical profibrotic stimuli, such as cardiac tissue-like cyclic mechanical stretching, could provide further insight on cardiac fibrosis development and treatment. In this regard, our substrates could be integrated into bioreactors or microfluidic systems supplying mechanical stretching.^28^

In conclusion, the results suggested that PCL/polyDOPA/C1F scaffolds triggered the activation of HCFs into myofibroblasts, synergistically integrating multiple mechanical, topological, and chemical cues peculiar of post-infarct environment, for the development of an *in vitro* model of human cardiac fibrosis. Furthermore, one additional advantage of the model is its potential future integration into mechanically stimulated microfluidic devices, providing miniaturized models for high-throughput preclinical testing.^51^

## 5 Conclusions

In this work, bioartificial PCL/polyDOPA/C1F electrospun scaffolds were designed to provide *in vivo*-like random nanofibrous architecture, surface composition, and stiffness with respect to post-infarct human cardiac fibrotic tissue. Such substrates were able to mimic: (i) the random organization of cardiac ECM components occurring during remodeling; (ii) the fibrotic cardiac ECM protein composition by the surface grafting of C1F (a blend of type I collagen and fibronectin); (iii) cardiac tissue stiffening occurring during early-stage fibrosis. Scaffolds supported the culture of HCFs, the cells mainly populating human cardiac fibrotic tissue. Particularly, PCL/polyDOPA/C1F membranes promoted cell adhesion, proliferation, and differentiation into myofibroblasts. The addition of TGF-β biochemical stimulus, which represents a key hallmark of cardiac fibrosis, did not cause significant differences in HCF behavior in terms of phenotype activation, demonstrating that the biomimetic chemical and physical properties of scaffolds were sufficient to trigger the cell phenotype switch into MyoFs.

Overall, the results demonstrated that PCL/polyDOPA/C1F membranes were suitable for the *in vitro* engineering of human cardiac fibrotic tissue, deserving future attention as *in vitro* tools for the preclinical validation of new therapies.

## Supporting Information Available

QCM-D analysis for polyDOPA/C1 and polyDOPA/F layer deposition: behavior of frequency shift and dissipation expressed as a function of time during deposition monitoring. Mass and thickness estimation for collagen type I, fibronectin, and C1F layer on polyDOPA precoating; AFM test on PCL membranes both in dry and wet conditions: Young’s modulus spatial distribution (PDF)
