# Supplementary material for: Biomimetic Electrospun Scaffold-Based In Vitro Model Resembling the Hallmarks of Human Myocardial Fibrotic Tissue
Source: ACS Biomater Sci Eng. 2023 Jun 8;9(7):4368–80. doi: 10.1021/acsbiomaterials.3c00483 (PMC10336747; doi:10.1021/acsbiomaterials.3c00483)
Supplement: Supplementary file 1 — ab3c00483_si_001.pdf [file ab3c00483_si_001.pdf]

Biomimetic electrospun scaffold-based *in vitro* model resembling the hallmarks of human myocardial fibrotic tissue

Gerardina RUOCCO [a, b, c], Alice ZOSO [a, b, c], Leonardo MORTATI [d], Irene CARMAGNOLA [a, b, c *], Valeria CHIONO [a, b, c *]

[a] Department of Mechanical and Aerospace Engineering, Politecnico di Torino, 10129 Torino TO, Italy

[b] POLITO Biomedlab, Politecnico di Torino, 10129 Torino TO, Italy

[c] Interuniversity Center for the promotion of the 3Rs principles in teaching and research, 56122 Pisa PI, Italy

[d] Istituto Nazionale di Ricerca Metrologica (INRIM), 10135 Torino TO, Italy.

*Co-last authors

Corresponding author: Valeria Chiono valeria.chiono@polito.it

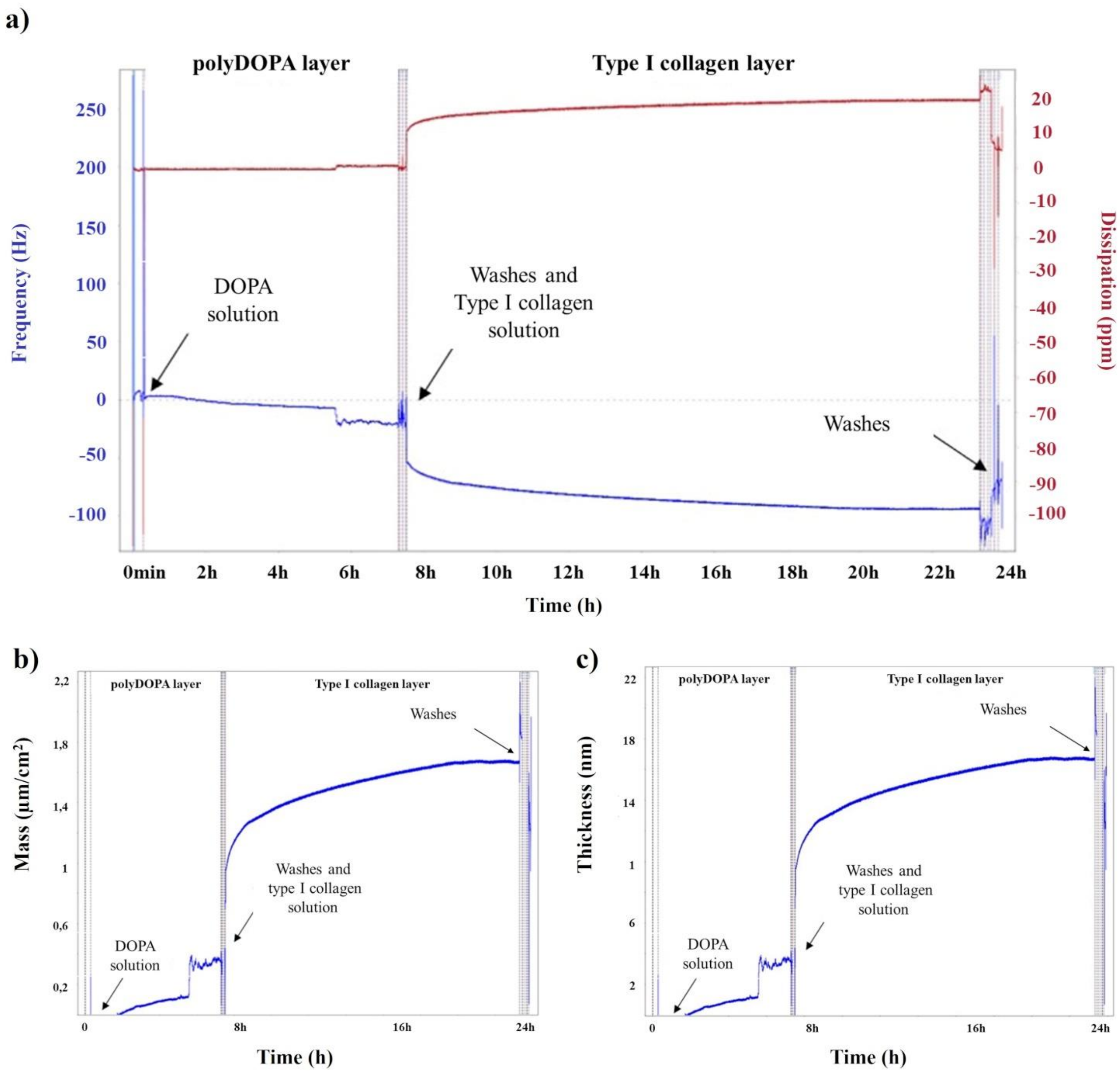

Figure S1 a) Behavior of frequency shift expressed in Hz (blue) and Dissipation expressed in ppm (red) as a function of time during the deposition of polyDOPA and type I collagen layers. Mass (b) and thickness (c) of polyDOPA and type I collagen layers as a function of time, estimated trough “Sauerbrey”(polyDOPA) and “Smartfit” (type I collagen) models respectively.

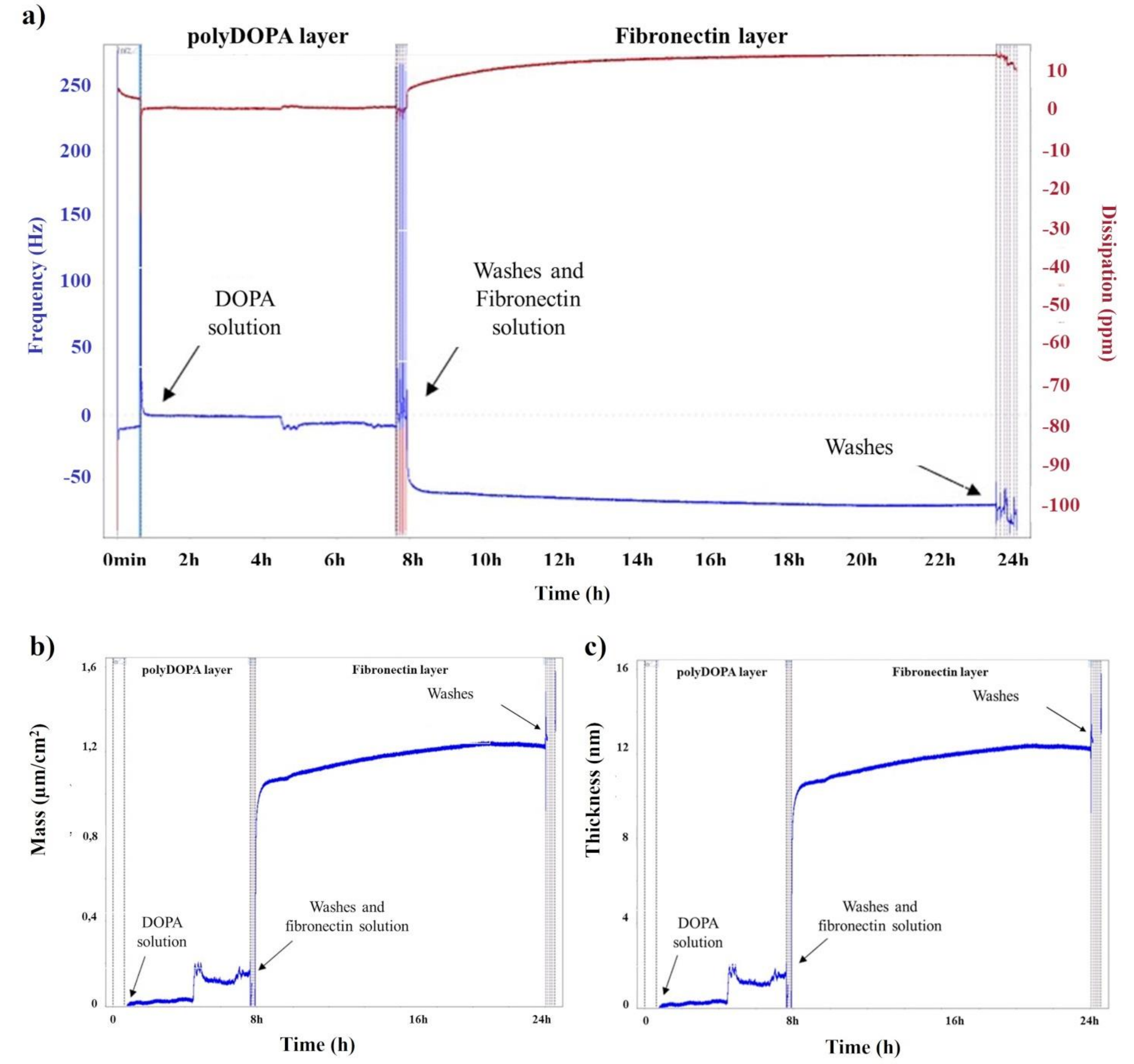

Figure S2 a) Behavior of frequency shift expressed in Hz (blue) and Dissipation expressed in ppm (red) as a function of time during the deposition of polyDOPA and fibronectin layers. Mass (b) and thickness (c) of polyDOPA and fibronectin layers as a function of time , estimated trough “Sauerbrey” (polyDOPA) and “Smartfit”(fibronectin) models respectively.

Mass and Thickness estimation

Grafted proteins	Mass (µg)	Thickness (nm)
Type I collagen	1.9	12
Fibronectin	1.7	11.4
C1F	5.5	35.8

Table S1 Mass and thickness for Collagen type I, Fibronectin and C1F layer on polyDOPA pre-coating.

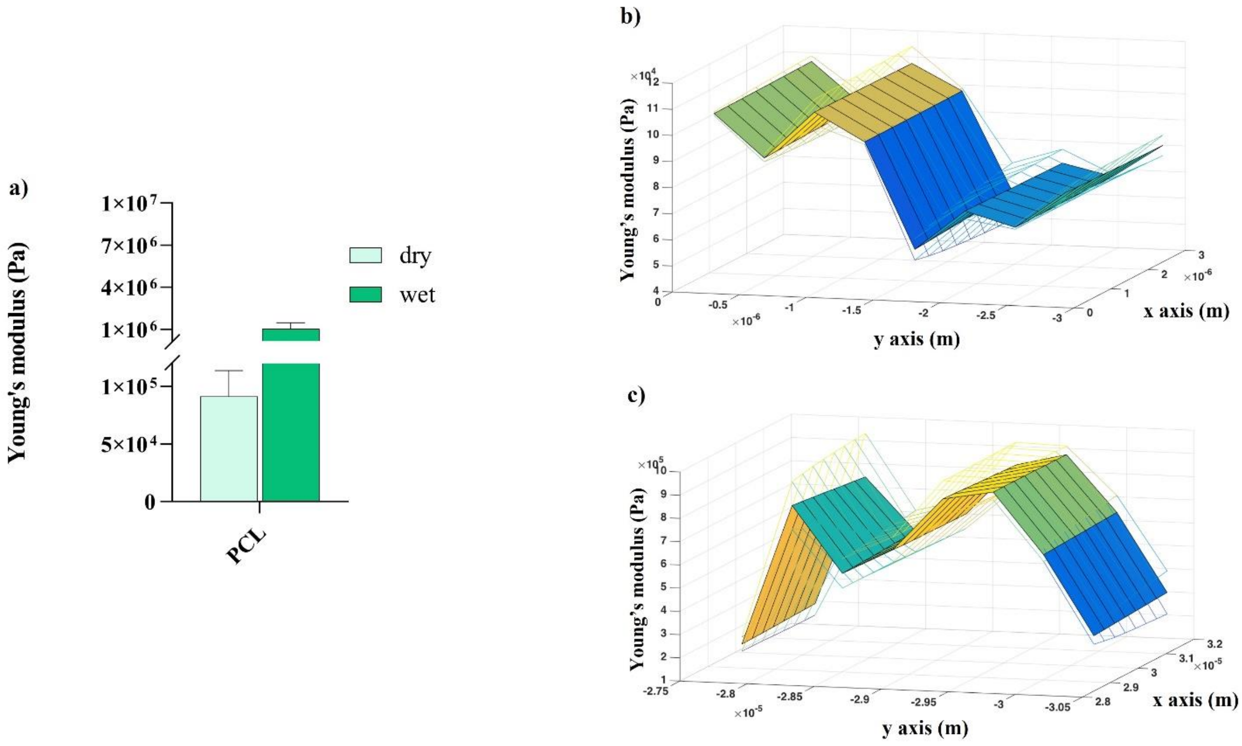

Figure S3 Young's modulus measured by AFM on PCL membranes in both dry and wet conditions. Average Young's modulus values obtained from all the grid points (a). Surface distribution of Young's modulus on PCL/polyDOPA/C1F membranes (in the selected experimental grid) in dry (b) and wet conditions (c).
